# Brain Pharmacokinetics and the Pharmacological Effects on Striatal Neurotransmitter Levels of *Pueraria lobata* Isoflavonoids in Rat

**DOI:** 10.3389/fphar.2017.00599

**Published:** 2017-09-05

**Authors:** Bingxin Xiao, Zengxian Sun, Fangrui Cao, Lisha Wang, Yonghong Liao, Xinmin Liu, Ruile Pan, Qi Chang

**Affiliations:** ^1^Institute of Medicinal Plant Development, Chinese Academy of Medical Sciences and Peking Union Medical College Beijing, China; ^2^Department of Clinical Pharmacology, The First People's Hospital of Lianyungang Lianyungang, China

**Keywords:** pharmacokinetic, *Pueraria lobata* isoflavonoids, neurotransmitters, microdialysis, UFLC-MS/MS

## Abstract

Isoflavonoids are putatively active components of *Pueraria lobata* and has been demonstrated prominent neuro-protection effect against cerebrovascular disorders, hypertension or Parkinson's disease (PD). However, the molecular basis for the beneficial effect of *Pueraria lobata* on nervous systems has not been well revealed. The present study aims to assess striatum exposure to main active isoflavonoids and changes of striatal extracellular neurotransmitters levels in rat brain after intravenous administration of *Pueraria lobata* isoflavonoids extracts (PLF), to further elucidate its' substantial bases for neuro activities. Fifteen rats were divided into 3 groups (five rats in each group) to receive a dose of PLF at 80 or 160 mg/kg or normal saline (vehicle), respectively. An LC-MS/MS method was employed to determine the concentrations of five main isoflavonoids and multiple neurotransmitters in microdialysate from striatal extracellular fluid (ECF) of the rats. The exposed quantities of puerarin (PU), 3′-methoxypuerarin (MPU), daidzein-8-C-apiosyl-(1-6)-glucoside (DAC), and 3′-hydroxypuerarin (HPU) in striatum were dose-dependent. The content of daidzein (DAZ) was too low to be detected in all dialysate samples through the experiment. Optimal dose PLF (80 mg/kg) promoted DA metabolism and inhibited 5-HT metabolism. No obvious change in the level of GLu was determined. The concentration of GABA presented a temporary decline firstly and then a gradual uptrend followed by a further downtrend. Higher dose (160 mg/kg) PLF could enhance the metabolism of both DA and 5-HT, and lower the extracellular level of GLu, without changing GABA concentrations, which might result in alleviation on excitatory toxicity under conditions, such as ischemia. The results infer that different dose of PLF should be chosen to achieve appropriate neurochemical modulation effects under conditions, such as hypertension or ischemia/stroke. These findings may significantly contribute to a better understanding of the neuroprotective effect of *Pueraria lobata* and provide new insights into its application toward neuro-degenerative diseases in the future.

## Introduction

Radix Puerariae, the dried root of *Pueraria lobata* (Willd.) Ohwi, has long been used as an ingredient of traditional Chinese prescriptions to treat fever, dysentery, certain types of cardiac/cerebral vascular diseases. The most abundant components of Radix Puerariae are isoflavones and isoflavone glycosides, which are aslo considered to be responsible for the therapeutic actions of Radix Pueraria under many conditions (Zhang et al., 2013). A wide range of pharmacological activities of the total isoflavones or major single component have been demonstrated, such as anti-inflammatory, anti-hypertension, spasmolytic effect, ameliorating microcirculation and protecting neurons against ischemia or neurodisorders (Lim et al., 2013; Li et al., 2017).

According to an animal study (Yan et al., 2004), *Pueraria lobata* isoflavones extracts (PLF) given orally exhibited antidepressant effect by reversing the pronounced low levels of norepinephrine (NE) and 4-dihydroxyphenylacetic acid (DOPAC, a metabolite of dopamine) in hippocampus or striatum of cerebral ischemia and reperfusion (CI/R) model mice. Moreover, as a major constituent of PLF, puerarin (PU), has been demonstrated potent protective effect against cortical neuron and astrocyte damage induced by oxygen-glucose deprivation or glutamate excitotoxicity *in vitro* (Xu and Zheng, 2007). In an *in-vivo* study, PU inhibited the ischemia-induced extracellular concentrations of aspartate, glutamate, amino butyric acid and taurine in striatum to alleviate the excitotoxicity in brain (Xu et al., 2007). As another component of PLF, 3'-methoxypuerarin (MPU), has also been found to increase the number of surviving neurons in hippocampa l region and markedly reduced the number of apoptotic pyramidal neurons after cerebral ischemia and reperfusion injury in rat brain(Liu et al., 2010). Thus, *Pueraria lobata* isoflavones can be viewed as promising agents for neuroprotection against ischemia or neurodisorders and the mechanism might involve the alleviation on excitotoxicity through the correction of neurotransmitter abnormalities. To our knowledge, there are kinds of neurotransmitters including amino acids, monoamines and peptides, keeping a relative balance in the brain, to regulate a variety of biological processes and behaviors. Abnormal releases and alteration in such a balance may lead to irreversible brain damage, which often happen in neuropathology and cerebral vascular diseases, such as depression, epilepsy, Parkinson's disease (PD), stroke and ischemia (Li et al., 2010). The determination of neurotransmitter dynamics in brain is essential for understanding the neuro-protective effect of *Pueraria lobata* isoflavones under conditions like ischemia or hypertension.

With the increasing significance of a potential beneficial role of *Pueraria lobata* isoflavones in neuron protection, as well as the increasing medications based on PLF in clinic, there is a growing demand for research into the pharmacokinetics (PK) profiles of major isoflavones from PLF in brain to understand the therapeutic basis and the compatibility rule of *Pueraria lobata* isoflavones for neuroactivities. In present study, we assessed the effect of acute systemic administration of PLF on extracellular levels of multiple neurotransmitters including dopamine (DA)/High vanillin aldehyde acid (HVA), glutamic acid (GLu)/Gamma Amino Acid Butyric Acid (GABA), 5-hydroxytryptamine (5-HT)/5-hydroxy indole acetic acid (5-HIAA), and acetylcholine (Ach) in striatum of freely moving rats and synthetically characterized the PK profiles of five compounds from PLF to further reveal the substantial basis and neurochemicals modulation mechanism of PLF for neuro activities.

## Materials and methods

### Chemicals and reagents

**Figure 1:**
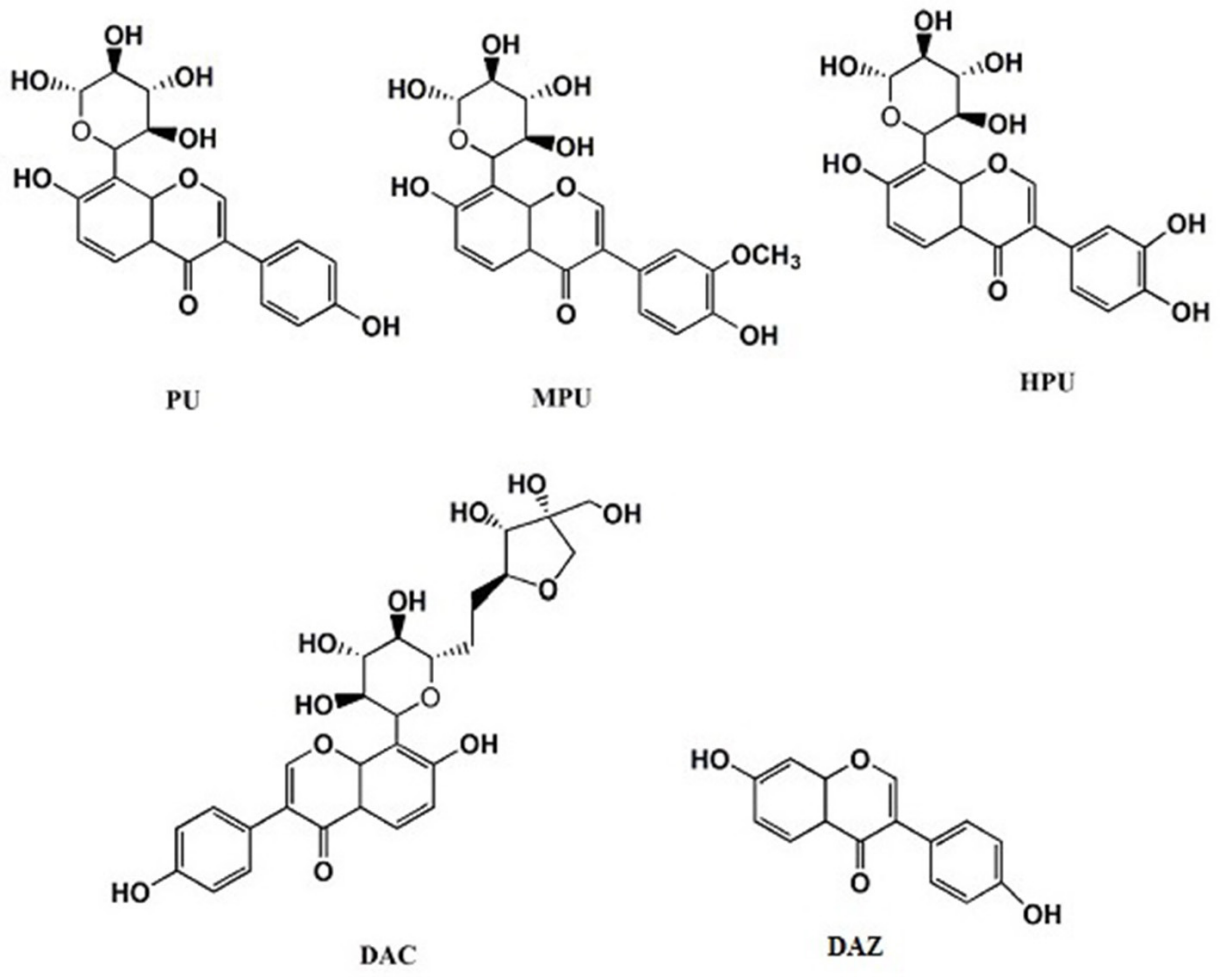
Chemical structures of the detected isoflavonoids, puerarin (PU), 3′-methoxypuerarin (MPU), 3′-hydroxypuerarin (HPU), daidzein (DAZ), and daidzein-8-c-apiosyl-(1-6)-glycoside (DAC).

The roots of *Puraria labata* were purchased from Beijing Tongrentang Pharmaceutical Company, (Beijing, China). The authentic standards of PU (98.40%), MPU (98.70%), 3′-hydroxypuerarin (HPU) (99.40%), daidzein (DAZ) (99.30%), and daidzein-8-C-apiosyl-(1-6)-glucoside (DAC) (92.1%) were prepared in our previous studies. The chemical structures of detected isoflavonoids are presented in Figure 1. Icarisid II (ICA) used as internal standard was purchased from Shanghai Winherb Medical Technology Company (ShangHai, China). The standards of DA (dopamine) and GLu (glutamic acid) were provided by the National Institutes for Food and Drug control (Beijing, China); The standards of 5-HT (5-hydroxytryptamine), HVA (High vanillin aldehyde acid), 5-HIAA (5-hydroxy indole acetic acid), Ach (acetylcholine), GABA (Gamma Amino Acid Butyric Acid) and Vitamin C, as well as DHBA (3, 4-2 hydroxy benzyl amine) used as the internal standard, were obtained from Sigma-Aldrich Co. Ltd. (Saint Louis, MO, USA). Acetonitrile and methanol with HPLC grade were purchased from Fisher Co.Ltd. (Emerson, USA). Formic acid and other reagents of analytical grade were purchased from Beijing Chemical Reagent Company (Beijing, China). Milli-Q water (18.3 MΩ; Milford, MA, USA) was used throughout the study.

### Preparation of PLF

The roots of *Puraria labata* were soaked with water three times. The extracted solutions were combined and loaded onto an absorption macroporous resin column (Type AB-8, The Chemical Plant of NanKai University, Tianjin, China). After loading, the column was washed with deionized water and then eluted with 70% ethanol. The 70% ethanol eluent was concentrated to dryness in vacuum to give a brown powder, namely PLF. The contents of the five isoflavonoids PU, MPU, HPU, DAZ, and DAC in PLF were determined as 27.90, 6.23, 5.78, 1.21, and 4.72% (w/w), respectively, by a reverse phase HPLC method with UV detection (Chi and Zhang, 2006; Xiao et al., 2016).

### Animals and surgeries

Male Sprague-Dawley rats (320 ± 10 g), supplied by Beijing Vital Laboratory Animal Technology (Beijing, China), were housed in a temperature (23 ± 2°C) and humidity controlled room maintained on a 12 h light/dark cycle with free access to food and water before the experiments. The experiment protocol was approved by the Animal Ethics Committee at the Institute of Medicinal Plant Development, Chinese Academy of Medical Sciences (SLXD-2015111976, 20 December 2015).

Before the experiments, 15 rats were randomly divided into three groups (five in each group). The rat was anesthetized by an intraperitoneal dose of chloral hydrate at 350 mg/kg and maintained by supplemental doses when required. The anesthetized rat was mounted in a stereotaxic apparatus (David Kopf, Tujunga, USA) on a flat-skull position. A midline incision was made through the skin over the upper side of the head to expose the skull and a hole was drilled in a dorsal position over the striatum at the following coordinates relative to bregma: Anterior (A): 0.6 mm; Lateral (L): 2.5 mm. A guide cannula [MAB6(9).14.IC; MAB, Stockholm, Sweden] was then lowered 3.5 mm ventral to dura surface through the hole and implanted into striatum with reference to the rat brain atlas of Paxinos and Watson (2005). It was anchored in place with screws and dental cement. Animals were allowed for at least 3 days recovery in separate cages. Prior to the experiment, the animal underwent the other minor surgery to cannulate a polyethylene tube (0.50 mm ID, 1.00 mm OD, Portex Limited, Hythe, Kent, England) into the right jugular vein under anesthesia as above method, for intravenous drug administration. It was then allowed recovery from the surgery for at least 24 h before the experiment.

### Microdialysis experiments

Before experiment, the microdialysis probe was pre-washed for 1 h with aCSF (147 mM NaCl, 4 mM KCl, 0.85 mM MgCl_2_, 2.3 mM CaCl_2_, pH 7.4) containing 20 units of heparin at a flow rate of 1 μL/min. The probe recovery (R_dial_) of each analyte was determined in advance, by using an *in vitro* method (Wang et al., 2008). The microdialysis experiment was conducted between 7:00 a.m. and 6:00 p.m. in a controlled environment. The rat was fasted for 12 h and first slightly anesthetized with diethylether to insert the probe into striatum through the guide cannula. Then the probe was connected to the sampling system comprised of a syringe micro infusion pump (CMA 400, CMA Microdialysis, Solna, Sweden), a freely moving equipment and a sample auto-collector (CMA 470, CMA Microdialysis, Solna, Sweden) set at 4^o^C. An equilibration period of 2 h perfusion with aCSF at 2.0 μL/min was allowed and the following 3 h of baseline samples were collected every 15 min time interval into the tubes containing 5 μL antioxidant (700 μg/mL vitamin C solution) before drug administration. PLF (80 or 160 mg/kg) were intravenously administrated to rats and dialysate samples were then collected every 15 min time interval for 3 h. Vehicle animals received normal saline solution (v/v, 1 mL/kg) by intravenous injection. The samples were stored at −80°C prior to analysis in 2 days.

### Sample treatment

An aliquot of 30 μL dialysate sample was spiked with 5 μL ICA solution (10 μg/mL) and 5 μL DHBA solution (50 μg/mL). The mixture was applied for vortex-mixing and then centrifugation at 4°C, 9,600 g/min for 15 min, and 10 μL of the supernatant was directly injected into the UFLC-MS/MS system for assay.

### UFLC-MS/MS conditions

A SHIMADZU Prominence UFLC system (Kyoto, JAPAN) connected with an Applied Biosystems 5500 Q-Trap mass spectrometer (Foster City, CA, USA) was used for simultaneous detection of the five compounds from PLF and seven neurotransmitters in intracephalic dialysate. The chromatographic separation was achieved by using a Luna C18 column (50 × 2.0 mm, 5 μm) from Phenomenex Company (San Jose, CA, USA). Gradient elution was employed using 0.05% formic acid in water (A) and acetonitrile (B) at a flow rate of 0.4 mL/min. The gradient elution program was as follows, 0–1.00 min (25% B); 1–3.50 min (25–70% B); 3.50–3.51 min (70–25% B); 3.51–4.50 min (25% B).

The mass spectrometer was operated with an electrospray ionization (ESI) interface in a positive/negative ion-switching mode. Multiple reactions monitoring (MRM) analysis was conducted by using the transitions of parent ions to product ions at *m/z* 415.0/267.1 (PU), 445.1/325.1 (MPU), 430.9/311.0 (HPU), 253.0/223.0 (DAZ), 547.2/295.0 (DAC), and 513.0/351.0 (ICA) in negative mode under ion spray voltage of -4500 V and *m/z* 148.2/84.0 (GLu), 104.0/87.0 (GABA), 146.0/87.4 (ACH), 177.0/160.0 (5-HT), 192.0/146.0 (5-HIAA), 183.0/136.9 (HVA), and 140.0/123.0 (DHBA) in positive mode under ion spray voltage of 5,500 V, respectively. The optimal working parameters of the interface were set as follows: curtain gas at 20 psi, nebulizer gas (Gas 1) at 40 psi, auxiliary gas (Gas 2) at 30 psi and a turbo ion spray temperature of 500°C. The ionization parameters for each compound are listed in Table 1. Data acquisition and procession was achieved using the Analyst 1.6.1 software version (AB SCIEX, Concord, Ontario, Canada).

**Table 1:** The ionization parameters for UFLC-MS/MS determination of five *P. lobata* isoflavonoids, seven neurotransmitters and IS (internal standard).

**Voltage(v)**	**DP**	**EP**	**CE**	**CXP**
PU	−240	−11	−46	−13
MPU	−210	−11	−34	−15
HPU	−260	−9	−34	−13
DAZ	−185	−15	−42	−11
DAC	−185	−14	−54	−13
ICA	−225	−15	−48	−21
GLu	95	4	17	9
GABA	50	10	17	8
Ach	160	10	22	30
5-HT	30	10	10	8
5-HIAA	133	13	17	8
DA	30	12	15	40
HVA	50	10	14	10
DHBA	60	5	7	15

*DP, Delustering Potential; EP, Entrance Potential; CE, Colliosion Energy; CXP, Collision Cell Exit Potential*.

### Data analysis

The analyte concentration (C) in brain striatum at each time point was calculated from its probe recovery (R_dial_) and determined concentration (C_d_) in dialysate by the equation, C = C_d_/R_dial_. The following pharmacokinetic parameters, peak concentration (C_max_), time to peak concentration (T_max_), area under the concentration-time curve from zero to time (AUC_0−t_) or infinity (AUC_INF_), apparent volume of distribution (Vd_λ*z*_/F), total body clearance (CL/F), mean residence time from zero to time (MRT_0−t_) or infinity (MRT_inf_) and half-life (t_1/2_) were estimated by analyzing drug concentration vs. time profile of each rat using non-compartmental model of WinNonlin software (Pharsight Corporation, Mountain View, CA, USA, Version 6.0).

### Statistical analysis

The One-way analysis of variance (ANOVA) was used for inter group comparisons of PK parameters. The two-way analysis of variance followed by Fisher's least significant test were used to test the statistical significance of difference between groups for pharmacological studies. The results are expressed as mean ± standard deviation (SD).

## Results and discussions

### Pharmacokinetics profiles of the five isoflavonoids in rat striatum

Before microdialysis experiment, the R_dial_ values of analytes were measured for every probe to calculate the concentrations in striatum. The mean R_dial_ values (*n* = 3) of PU, MPU, HPU, DAZ, and DAC perfusated with aCSF were determined as 20.61, 21.48, 22.78, 14.70, and 11.53%, respectively, under the experimental conditions. In this study, PK profiles of PU, MPU, HPU, and DAC were evaluated in rat striatum after intravenous administration of PLF at 80 or 160 mg/kg. The content of DAZ was too low to be detected in all dialysate samples through the experiment, which might be obstructed by the blood-brain barrier.

**Figure 2:**
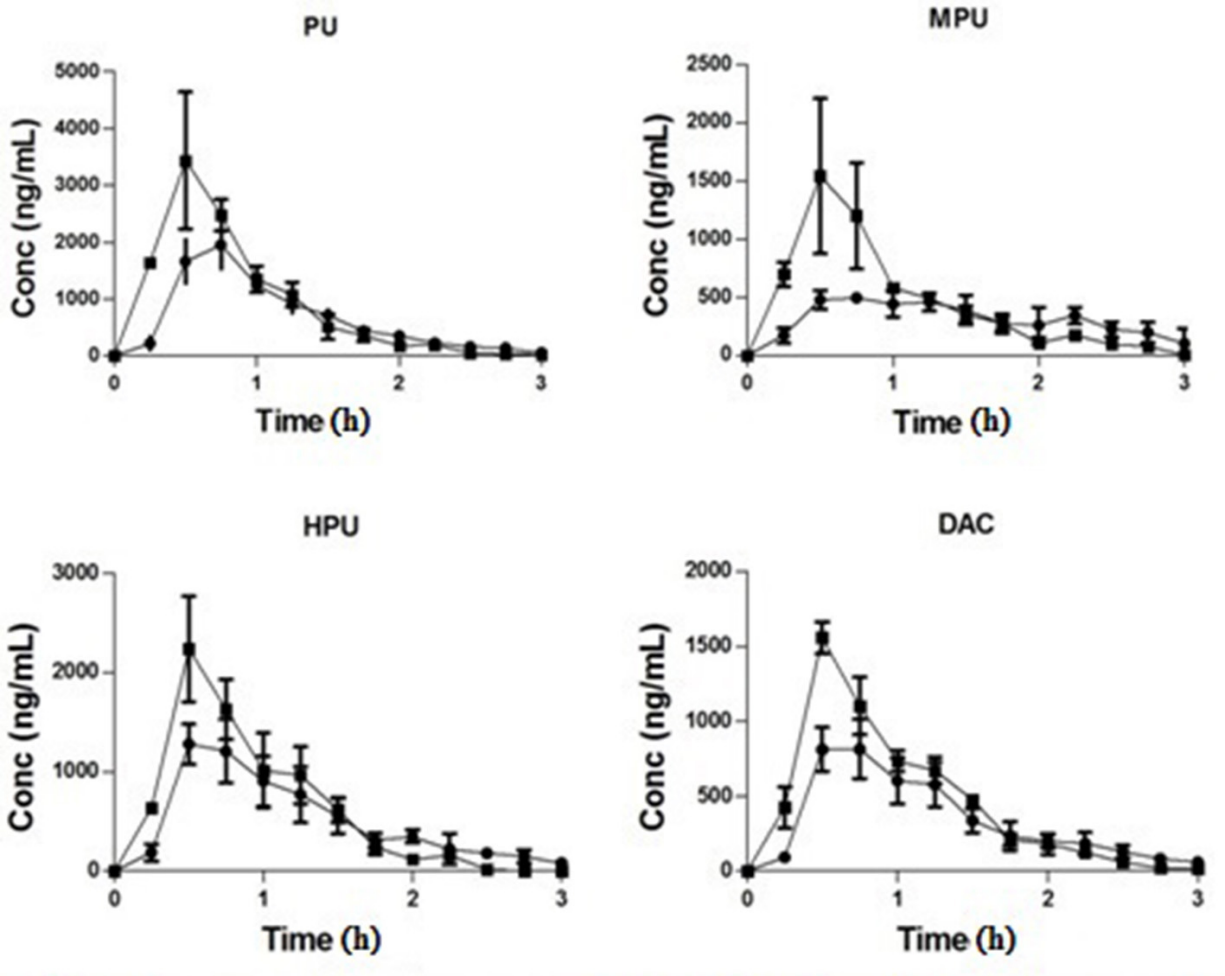
Concentration-time curve of PU, MPU, HPU, and DAC in rat stratium after intravenous administration of PLF at 80 (♦) and 160 (■) mg/kg.

The striatum concentration vs. time courses of PU, MPU, HPU, and DAC are presented in Figure 2. The estimated pharmacokinetic parameters are presented in Table 2. Following 80 mg/kg dose of PLF, the concentrations of PU, MPU, HPU, and DAC in striatum increased gradually and reached the peak concentration of 2.19, 0.65, 1.30, and 1.02 μg/mL at about 0.95 h. The results indicated that the four isoflavonoids can penetrate through the blood–brain barrier (BBB) quickly, which are consistent with the reported studies (Yan et al., 2006; Yang et al., 2014). Their elimination from the brain was relatively rapid with t_1/2_ at 0.30, 0.99, 0.34, and 0.29 h, respectively. The average AUC_0−t_ value of the four compounds was determined as 2.14, 1.02, 1.40, and 1.11 μg^*^h/mL, respectively. The MRT of PU, MPU, HPU, and DAC in striatum was calculated as 1.21, 2.34 0.17, and 0.82 h. Compared to data from our previous studies (Xiao et al., 2016), the C_max_ of PU, MPU, and DAC in striatum extracellular fluid (ECF) was higher than that observed in ventricular CSF (cerebrospinal fluid) by a multiple of 4.20, 1.56 and 2.68, as well as AUC_0−t_ which represents 3.06, 1.85, and 3.17 folds higher value than that in CSF, respectively after administration of 80 mg/kg PLF. The distribution of PU and MPU from CSF to striatum may take about 0.17 and 0.82 h as the MRT of PU and MPU in striatum was that longer than it in CSF, while the distribution of DAC is quick with the close MRT value in striatum and CSF. A previous study (Wei et al., 2015) demonstrated that PU had a higher concentration and longer retention time in neurons than that observed in astrocytes. We therefore concluded that differences exist in PK profiles of compounds in distinct brain region attributing to the heterogeneity of CNS circulatory system. The phenomenon should be considered in evaluating the concentration and distributional processes that mediate delivery of drug molecules to their pharmacological or toxicological sites of action in brain.

**Table 2:** Pharmacokinetic parameters of PU, MPU, HPU, and DAC in rat striatum following intravenous administration of PLF at 80 and160 mg/kg, respectively.

**PK parameters**	**PU**		**MPU**		**HPU**		**DAC**	
**Dose(mg/kg)**	**80**	**160**	**80**	**160**	**80**	**160**	**80**	**160**
T_max_ (h)	0.95 ± 0.31	0.50 ± 0.12	0.95 ± 0.41	0.65 ± 0.28	0.95 ± 0.41	0.65 ± 0.28	0.95 ± 0.41	0.70 ± 0.20
C_max_ (μg/mL)	2.19 ± 0.39	4.13 ± 1.48^*^	0.65 ± 0.04	1.44 ± 0.51^*^	1.30 ± 0.24	2.07 ± 0.68^*^	1.02 ± 0.32	2.13 ± 0.89^**^
t_1/2, λ_(h)	0.30 ± 0.19	0.20 ± 0.17	0.99 ± 0.11	0.20 ± 0.08^*^	0.34 ± 0.17	0.16 ± 0.07	0.29 ± 0.16	0.24 ± 0.12
AUC_0−t_(μg·h/mL)	2.14 ± 0.33	3.19 ± 0.42^*^	1.02 ± 0.04	1.35 ± 0.26^*^	1.40 ± 0.28	2.10 ± 0.67	1.11 ± 0.22	1.63 ± 0.32^*^
AUC_inf_(μg·h/mL)	2.20 ± 0.36	3.24 ± 1.38	1.23 ± 0.24	1.35 ± 0.25	1.48 ± 0.34	2.43 ± 0.64	1.15 ± 0.24	1.65 ± 0.34
CL/F (L/h/kg)	10.34 ± 1.79	19.71 ± 5.39	4.16 ± 0.21	6.86 ± 1.74	2.64 ± 0.89	4.79 ± 1.76	3.39 ± 0.77	5.36 ± 1.33
V _dλz_/F (L/kg)	4.49 ± 2.35	4.29 ± 0.82	8.98 ± 2.75	2.00 ± 0.42	1.60 ± 0.74	1.04 ± 0.69	1.41 ± 0.77	1.60 ± 0.89
MRT_0−t_(h)	1.15 ± 0.25	0.80 ± 0.09	1.28 ± 0.28	0.87 ± 0.21	1.20 ± 0.28	0.79 ± 0.14	1.28 ± 0.28	0.94 ± 0.21
MRT_inf_(h)	1.21 ± 0.29	0.82 ± 0.11	2.34 ± 0.58	0.89 ± 0.23	1.29 ± 0.38	0.83 ± 0.09	1.37 ± 0.37	1.02 ± 0.37

*All data are expressed as Mean ± SD (n = 5)*.

Compared inter groups. (

* *P < 0.05*,

** *P < 0.01)*.

Along with dose increment from 80 to 160 mg/kg, the C_max_ of PU, MPU, HPU and DAC in striatum was increased to 4.13, 1.44, 2.07, and 2.13 μg/mL at a shorter T_max_ of 0.50, 0.65, 0.65 and 0.70 h, while AUC_0−t_ increased to 3.19, 1.35, 2.10, and 1.63 μg^*^h/mL. The MRT of the four compounds were significantly decreased to 0.82, 0.89, 0.83, and 1.02 h. The t_1/2_ of PU, MPU, HPU, and DAC were calculated as 0.20, 0.20, 0.16, and 0.24 h. The results showed that penetration contents of the four main compounds in striatum were increased according to dose increment. In order to increase steady-state concentration of active compounds in target pharmacological site of brain, higher dose of PLF should be chosen.

### Changes in extracellular transmitter levels in striatum and their potential consequences

**Figure 3:**
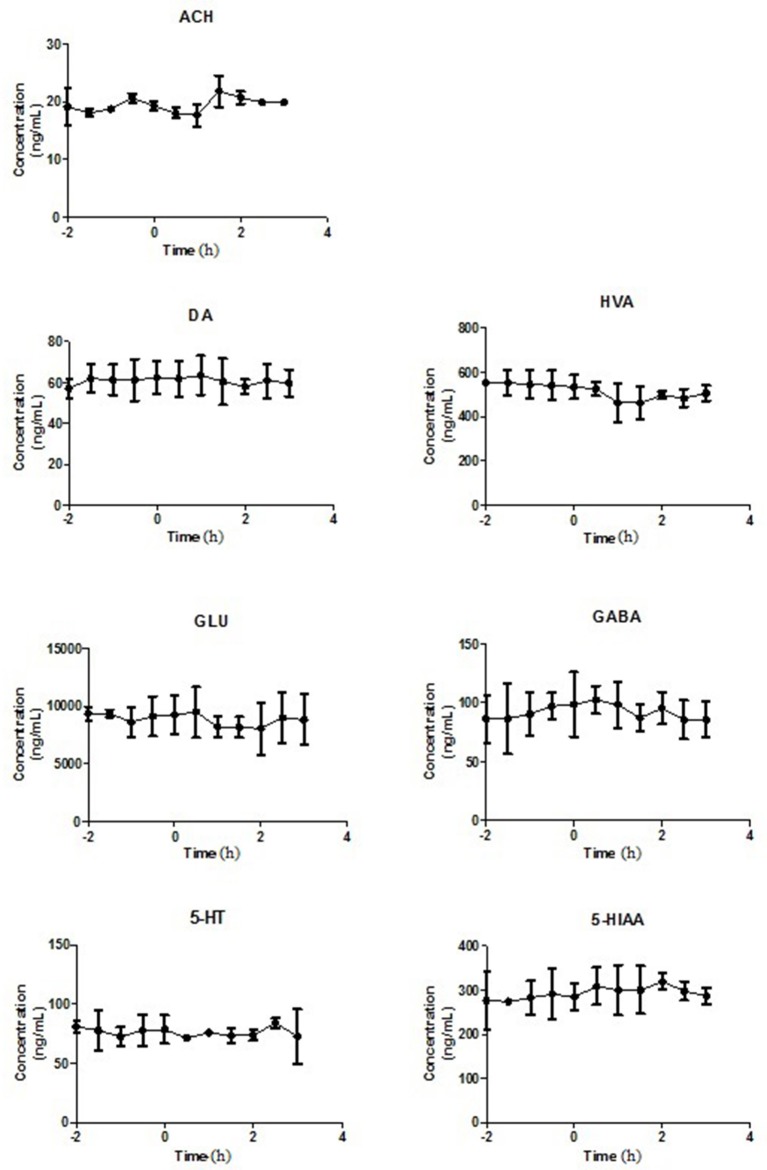
The levels of neurotransmitters in extracellular fluid of striatum of rats after intravenous administration of Normal Saline. Data are expressed as Mean ± SD (*n* = 5).

**Figure 4:**
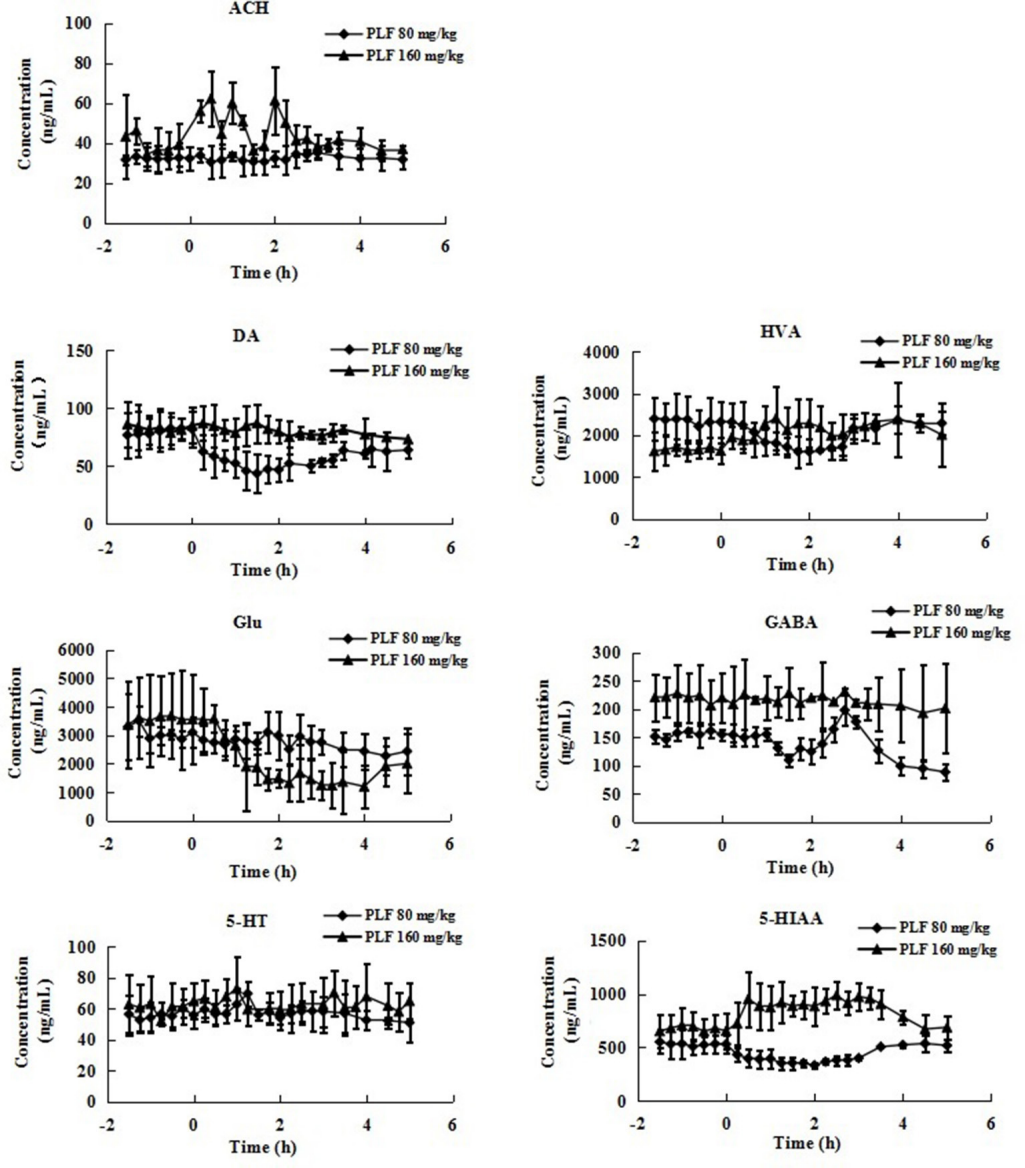
The levels of neurotransmitters in extracellular fluid of striatum of rats after intravenous administration of PLF at a dose of 80 or 160 mg/kg. Data are expressed as Mean ± SD (*n* = 5).

In present study, the mean R_dial_ values (*n* = 3) of DA, HVA, GLu, GABA, 5-HT, 5-HIAA and Ach were also evaluated as 22.59, 34.63, 33.05, 31.11, 25.54, 27.76, and 10.74%, respectively. The basal levels of above transmitters in striatum were 81.87 ± 15.16, 2320.78 ± 489.04, 3097.42 ± 519.40, 154.88 ± 10.11, 55.94 ± 8.42, 528.96 ± 88.06, and 32.34 ± 5.65 ng/mL respectively (Table 3). The effects of vehicle saline or PLF (80 and 160 mg/kg i.v. administration) on striatum transmitter levels of freely moving rats are elucidated in Figure 3 or Figure 4. Significant decreases of DA and HVA levels were found by the maximum percentage of 46.6 and 30.6% at 1.5 and 1.75 h, respectively, after 80 mg/kg dose of PLF. The HVA/DA ratio was increased from 28.35 to the maximum of 39.38 at 1.25 h post dose; this indicated that the metabolism of dopaminergic neuro chemicals might be promoted. No significant change of GLu level was observed. The level of GABA presented a temporary decline firstly, and then gradually increased to the maximum concentration of 197 ng/mL at 3 h, followed by a downtrend until 5 h after dose. The level of 5-HT was not affected, while the level of 5-HIAA decreased immediately and reached the minimal concentration of 331 ng/mL at 2 h after dose. These results suggested that the metabolism of serotonergic neurotransmitters is inhibited by the treatment of PLF at 80 mg/kg. No significant change was detected in the level of Ach in striatum.

**Table 3:** The basal and peak levels of neuro chemicals in rat brain from PLF (80 or 160 mg/kg) treated or vehicle group.

**Group**	**1 (80 mg/kg)**		**2 (160 mg/kg)**		**3 (Vehicle)**		**Statistics**		
	**Concentration (ng/mL)**								
**Neuro chemical**	**Basal level (before dose)**	**Peak level (post dose)**	**Basal level (before dose)**	**Peak level (post dose)**	**Basal level (before dose)**	**Peak level (post dose)**	**df**	**F**	**P**
DA	81.87 ± 15.16	46.88 ± 7.16^**^	85.07 ± 15.06	75.16 ± 14.29	62.38 ± 8.16	58.03 ± 3.52	2	44.96	<0.001
HVA	2320.78 ± 489.04	1611.73 ± 370.66^*^	1633.95 ± 300.06	2405.03 ± 433.63^*^	535.32 ± 53.71	462.38 ± 71.74	2	247.38	<0.001
5-HT	55.94 ± 8.42	69.79 ± 8.07	64.99 ± 11.42	73.10 ± 20.61	78.51 ± 11.94	84.12 ± 4.25	2	141.16	<0.001
5-HIAA	528.96 ± 88.06	331.72 ± 28.17^**^	653.41 ± 162.77	987.40 ± 125.32^**^	284.91 ± 31.21	319.84 ± 18.82	2	156.68	<0.001
GLu	3097.42 ± 519.40	2278.02 ± 647.76	3564.58 ± 1558.16	1202.71 ± 762.13^**^	9224.44 ± 1658.01	8035.41 ± 2245.20	2	770.95	<0.001
GABA	154.88 ± 10.01	194.84 ± 27.59^*^	220.20 ± 43.13	225.95 ± 61.95	98.70 ± 27.42	85.62 ± 16.40	2	406.29	<0.001
ACH	32.34 ± 5.65	30.65 ± 6.29	39.23 ± 10.92	62.29 ± 14.10^*^	19.24 ± 0.81	21.81 ± 2.69	2	133.385	<0.001

*All data are expressed as Mean ± SD (n = 5).The main effect df, F and P-value were obtained through two-way analysis of variance followed by Fisher's least to test the statistical significance of difference between groups*.

Compared to basal level (

* *P < 0.05*,

** *P < 0.01)*.

It was also demonstrated that at a higher dose of 160 mg/kg, PLF could increase the extracellular levels of HVA and 5-HIAA and do not affect the levels of DA and 5-HT in striatum. The ratios of HVA/DA and 5-HIAA/5-HT were increased; this indicated that the metabolism of DA and 5-HT is enhanced. Moreover, PLF could lower the extracellular level of GLu, of which the concentration decreased from the basal level of 3,564 ng/mL to the minimum of 1,250 ng/mL by a percentage of 64.93% at 3 h post dose, while the level of GABA was not changed. Ach level presented a wavy curve with an up going trend in 0–5 h after dose.

It is well recognized that the excessive releases of GLu can provoke enzymatic process leading to irreversible neuronal injury (Guyot et al., 2001; Li and Yan, 2010). Some evidences indicate that the excessive release of DA and 5-HT may involve the generation of cytotoxic free radicals and aggravate excitotoxicity of excitatory amino acid (Cadet et al., 2000; Yoshimoto et al., 2014). It has been reported that the pretreatment of puerarin could attenuate glutamate-induced cell injury in a dose-dependent manner (Zhu et al., 2016). The administration of PU could inhibit the effect of 5-HT by activating 5-HT1 receptor and/or antagonizing 5-HT2A receptors in the hypothalamus (Chueh et al., 2004). And it has been revealed that long-term PU treatment can restore the contents of dopamine and its metabolites against Parkinson's disease (Zhu et al., 2010). The mechanism might invlove upregulation on the vesicular monoamine transporter 2 (Zhang et al., 2014). So we conclude that the modulation on the concentration of GLu/GABA, 5-HT, DA, and the metabolites might be one of the mechanism for neuroprotection activities of PLF.

Our experiment results indicated that PLF could decrease the concentration or promote the metabolism of GLu, DA and 5-HT in striatum at 160 mg/kg dose, which could form a protective mechanism and relieve the excite toxic neuronal injury against some cerebrovascular or neuro diseases. Toward the neuroprotection effects mediated by alleviation on excitatory toxicity, a higher dose of PLF is recommended.

## Conclusion

In this study, the PK profiles of five isoflavonoids from PLF in striatum were determined. The results indicated that PU, MPU, HPU, and DAC can penetrate through the blood brain barrier quickly and achieve high concentrations in striatum after intravenous dose with PLF. The exposed quantities were dose-dependent. PLF presents different modulation effects on neurotransmitters at different doses, which infer that different dose of PLF should be chosen to achieve appropriate neuro chemical modulation effects under conditions, such as hypertension or stroke. These findings may significantly contribute to a better understanding of the neuroprotective effect of *Pueraria lobata* and provide new insights into its application toward neuro-degenerative diseases in the future.

## Author contributions

QC, BX, RP, YL, and XL participated in research design. BX, ZS, and LW performed the experiments and data analysis. QC, BX, ZS, and FC contributed to the writing of the manuscript.

### Conflict of interest statement

The authors declare that the research was conducted in the absence of any commercial or financial relationships that could be construed as a potential conflict of interest.

## References

[B1] Cadet J. L. Harrington B. Ordonez A. S. (2000). Bcl-2 overexpression attenuates dopamine-induced apoptosis in an immortalized neural cell line by suppressing theproduction of reactive oxygen species. Synapse 35, 228–233. 10.1002/(sici)1098-2396(20000301)35:3 10657030

[B2] Chi J. F. Zhang G. G. (2006). Determination of Puerarin in Radix Puerariae by HPLC. Zhong Nan Yao Xue. 4, 307–309. 10.3969/j.issn.1672-2981.2006.04.028

[B3] Chueh F. S. Chang C. P. Chio C. C. Lin M. T. (2004). Puerarin acts through brain serotonergic mechanisms to induce thermal effects. J. Pharmacol. Sci. 96, 420–427. 10.1254/jphs.FP0040424 15599109

[B4] Guyot L. L. Diaz F. G. O'Regan M. H. McLeod S. (2001). Real-time measurement of glutamate release from the ischemic penumbra of the rat cerebral cortex using a focal middle cerebral artery occlusion model. Neurosci. Lett. 299, 37–40. 10.1016/S0304-3940(01)01510-5 11166932

[B5] Li H. Li C. Yan Z. Y. Yang J. Chen H. (2010). Simultaneous monitoring multiple neurotransmitters and neuromodulators during cerebral ischemia/reperfusion in rats by microdialysis and capillary electrophoresis. J. Neurosci. Methods. 189, 162–168. 10.1016/j.jneumeth.2010.03.022 20347872

[B6] Li H. Yan Z. Y. (2010). Analysis of amino acid neurotransmitters inhypothalamus of rats during cerebral ischemia-reperfusion by microdialysis and capillary electrophoresis. Biomed. Chromatogr. 24, 1185–1192. 10.1002/bmc.1425 20954209

[B7] Li S. Li S. Liu C. Y. Liu C. Zhang Y. C. (2017). Extraction and isolation of potential anti-stroke compounds from flowers of *Pueraria lobata* guided by *in vitro* PC12 cell model. J. Chromatogr. B Analyt. Technol. Biomed. Life Sci. 1048, 111–120. 10.1016/j.jchromb.2017.02.009 28236683

[B8] Lim D. W. Lee C. Kim I. H. Kim Y. T. (2013). Anti-inflammatory effects of total isoflavones from *Pueraria lobata* on cerebral ischemia in rats. Molecules 18, 10404–10412. 10.3390/molecules180910404 23989686 PMC6270189

[B9] Liu Y. Chen X. Liu K. Chai Q. Li J. Liu Z.. (2010). Protection by 3'-methoxypuerarin of rat hippocampal neurons against ischemia/reperfusion injury. Chin. J. Physiol. 53, 136–140. 10.4077/CJP.2010.AMK029 21793321

[B10] Paxinos G. Watson C. (2005). The Rat Brain in Stereotaxic Coordinates, New York, NY: Elsevier Academic Press.

[B11] Wang Q. Chen G. Zeng S. (2008). Distribution and metabolism of gastrodin in rat brain. J. Pharm. Biomed. Anal. 46, 399–404. 10.1016/j.jpba.2007.10.017 18053670

[B12] Wei S. Y. Tong J. Xue Q. Shang F. H. Li Y. J. Liu Y.. (2015). Puerarin exhibits greater distribution and longer retention time in neurons than astrocytes in a co-cultured system. Neural Regen Res. 10, 605–609. 10.4103/1673-5374.155435 26170822 PMC4424754

[B13] Xiao B. X. Feng L. Cao F. R. Pan R. L. Liao Y. H. Liu X. M.. (2016). Pharmacokinetic profiles ofthe five isoflavonoids from *Pueraria lobata* roots intheCSF and plasma of rats. J. Ethnopharmacol. 184, 22–29. 10.1016/j.jep.2016.02.027 26923541

[B14] Xu X. H. Zheng X. X. Zhou Q. Li H. (2007). Inhibition of excitatory amino acid efflux contributes to protective effects of puerarin against cerebral ischemia in rats. Biomed. Environ. Sci. 20, 336–342. 17948770

[B15] Xu X. Zheng X. (2007). Potential involvement of calcium and nitric oxide in protective effects of puerarin on oxygen-glucose deprivation in cultured hippocampal neurons. J. Ethnopharmacol. 113, 421–426. 10.1016/j.jep.2007.06.012 17698307

[B16] Yan B. Wang D. Y. Xing D. M. Ding Y. Wang R. F. Lei F.. (2004). The antidepressant effect of ethanol extract of radix puerariae in mice exposed to cerebral ischemia reperfusion. Pharmacol. Biochem. Behav. 78, 319–325. 10.1016/j.pbb.2004.04.010 15219773

[B17] Yan B. Wang W. Zhang L. Xing D. Wang D. Du L. (2006). Determination of puerarin in rat cortex by high-performance liquid chromatography after intravenous administration of Puerariae flavonoids. Biomed. Chromatogr. 20, 180–184. 10.1002/bmc.549 16078309

[B18] Yang Y. Bai L. Li X. Xiong J. Xu P. Guo C.. (2014). Transport of active flavonoids based on cytotoxicity and lipophilicity: an evaluation using the blood-brain barrier cell and Caco-2 cell models. Toxicol. In Vitro 28, 388–396. 10.1016/j.tiv.2013.12.002 24362044

[B19] Yoshimoto K. Namera A. Arima Y. Nagao T. Saji H. Takasaka T.. (2014). Experimental studies of remarkable monoamine releases and neural resistance to the transient ischemia and reperfusion. Pathophysiology 21, 309–316. 10.1016/j.pathophys.2014.08.005 25270870

[B20] Zhang X. Xiong J. Liu S. Wang L. Huang J. Liu L.. (2014). Puerarin protects dopaminergic neurons in Parkinson's disease models. Neuroscience 280, 88–98. 10.1016/j.neuroscience.2014.08.052 25218963

[B21] Zhang Z. Lam T. N. Zuo Z. (2013). Radix Puerariae: an overview of its chemistry, pharmacology, pharmacokinetics, and clinical use. J. Clin. Pharmacol. 53, 787–811. 10.1002/jcph.96 23677886

[B22] Zhu G. Q. Wang X. C. Chen Y. F. Yang S. Cheng H. Wang N.. (2010). Puerarin protects dopaminergic neurons against 6-hydroxydopamine neurotoxicity via inhibiting apoptosis and upregulating glial cell line-derived neurotrophic factor in a rat model of Parkinson's disease. Planta Med. 76, 1820–1826. 10.1055/s-0030-1249976 20509103

[B23] Zhu X. Wang K. Zhang K. Lin X. F. Zhu L. Zhou F. F. (2016). Puerarin protects human neuroblastoma SH-SY5Y cells against glutamate-induced oxidative stress and mitochondrial dysfunction. J. Biochem. Mol. Toxicol. 30, 22–28. 10.1002/jbt.21736 26277993

